# Evaluating the compatibility of the 2D and 3D facial soft tissue depth measurement methods

**DOI:** 10.1007/s00414-025-03585-0

**Published:** 2025-08-27

**Authors:** Gülçin Coşkun

**Affiliations:** School of History, Classics& Archaeology, University of Edinburgh, William Robertson Wing, Teviot Place, Edinburgh, EH8 9AG UK

**Keywords:** Forensic facial approximation, Facial soft tissue depth, CT image analysis, Image segmentation, Virtual anthropology, Amira

## Abstract

Forensic facial approximation is a technique that involves constructing the facial muscles and applying a suitable facial soft tissue depth (FSTD) dataset. To date, several FSTD studies have been conducted for varying population groups by using different FSTD measurement methods (i.e., needle puncture, two-dimensional (2D) and three-dimensional (3D) FSTD measurement methods). This study aims to compare the 2D and 3D FSTD measurement methods. The facial depths of subjects were measured using 100 head CT scans of 50 male and 50 female subjects aged between 18 and 99. For the 3D method, the 3D head and skull models of individuals were created by using the Histogram method to segment the CT images in Amira 6.1. Subsequently, the 3D depth measurements were obtained from the 3D head and skull models at 15 (4 mid-sagittal and 11 bilateral) cranial landmarks. For the 2D method, the FSTD measurements were directly taken from the CT slices at the same cranial landmarks as the 3D method. The intra- and inter-observer errors of the measurements were assessed using a *repeated measures MANOVA*. Similarly, the discrepancies between the FSTD datasets obtained using the 2D and 3D methods were evaluated using a *repeated measures MANOVA*. The depth differences between the two FSTD datasets were statistically significant, yet they were negligible from a practical point of view.

## Introduction

Forensic facial approximation is a method to assist in the identification of skeletonised human remains when the positive (e.g., DNA analysis and the comparison of dental records) and presumptive (e.g., biological profile) identification methods are insufficient in pointing to an identity [1–3]. The method has been performed throughout human history for different purposes, such as teaching anatomy, identifying unknown skulls and recreating the faces of historical characters by applying various materials (e.g., clay, wax, mastic, plasticine, etc.) over the skull [4]. Facial approximation can be achieved through three distinct techniques: two-dimensional (2D), three-dimensional (3D) manual and computer-based methods [5, 6]. The two-dimensional facial approximation involves drawing the faces of unidentified skulls on paper, incorporating details of facial features and the depth of facial soft tissues into the approximation [7].

The 3D manual method involves three primary approaches: the Russian, American and Combination methods [6, 8–12]. Mikhail Mikhaylovich Gerasimov (1907–1970), a Russian archaeologist and anthropologist, introduced an anatomical perspective to facial approximation by developing what is known as the Russian method (also referred to as morphoscopic or anatomical method) [8]. Contrary to earlier approximation examples, Gerasimov’s approach [8] included facial musculature. He achieved facial approximation by applying plasticine over the musculature to represent the skin. It is significant to note that there was a misunderstanding in the early literature available in English [5, 6, 9], which indicated that Gerasimov reconstructed all muscles of facial expression and mastication. This misunderstanding was clarified by Ullrich and Stephan [13]. They pointed out that Gerasimov only reconstructed two specific masticatory muscles—the temporalis and masseter—on each side of the face. The other common fallacy is that Gerasimov did not incorporate FSTD in his facial approximations. In fact, he established his soft tissue depth dataset using 71 cadavers [13] and used the FSTD tables belonging to populations from the former USSR [14]. Moreover, he used the wax pyramids as the depth markers in facial approximations, representing the facial soft tissue depths [14]. This indicates that the Russian method (1955) relies on facial soft tissue depth data [13].

The American method, known as the morphometric or anthropometric method, was developed by Wilton Krogman (1903–1988) and his collaborators [6, 12, 15]. This method involves applying facial soft tissue depth measurements to a skull cast, taking into account the individual’s age, sex and population affinity [6, 10, 11, 16]. While the facial muscles are not individually reconstructed in the American method, anatomical knowledge is still incorporated when applying the facial soft tissue depths [5].

The Combination Method, also known as the Manchester method, was developed by Richard Neave (1936-), a British medical artist [5, 6, 12]. This method integrates the anatomical approach of the Russian Method with the soft tissue depth application of the American Method. Unlike the Russian Method, the Combination Method reconstructs all superficial and deep muscles involved in facial expression, mastication, and neck movements. Although three different manual approximation techniques have been suggested, the Combination Method is widely accepted within the field [6].

The computer-based method is a 3D digital facial approximation technique that was suggested as an alternative to the 3D manual method. This approach aims to reduce the subjective interpretations of practitioners while providing a more time-efficient solution [16–18]. The method can be categorized into two types: automated and non-automated [16, 17, 19]. The automated method is achieved by fitting facial templates obtained from living individuals onto the skull of interest by warping these templates. In contrast, the non-automated method mimics the 3D manual methods [5, 17, 19, 20].

FSTD data plays a key role when reconstructing a face, regardless of the facial approximation methods employed. To date, several FSTD studies have been conducted across various population groups using different measurement methods [3]^21^– [32–34]. The first FSTD study was conducted by Hermann Welcker in 1883 [35]. He measured the depth from the nine midline landmarks of adult cadavers using a double-edged knife. Following Welcker, Wilhelm His [36] measured the facial depth of cadavers using a sewing needle with a rubber disk. In this new method, which is referred to as the needle puncture method, the needle penetrated the skin at the predetermined point of the face till it reached the bone. The rubber disk was then lowered up to the surface of the skin by refraining from putting pressure on the skin. The length between the tip of the needle and where the rubber disk stopped gave the facial depth of a given landmark [6, 36]. This needle puncture method remains popular among researchers due to its cost-effectiveness and straightforward measurement equipment [22]. However, this method has been criticised because the needle exerts pressure on the soft tissue as it penetrates the skin, leading to soft tissue depth distortion. Additionally, accurately identifying the cranial landmarks from the surface of the skin can be complex [22, 37, 38]. The major limitation of this method is the distortion caused by postmortem changes [30, 39]. Researchers have proposed various solutions to address this issue, such as taking measurements within the first 12 [39, 40] or 24 h after death [32, 41], or embalming the cadavers [42].

Improvements in the medical image modalities, such as radiograph, ultrasound, MRI, CT and CBCT, have enabled researchers to obtain the FSTDs from living individuals. Several studies [21, 24, 30]^43^– [34, 45] utilized these advanced imaging technologies to create datasets using the two-dimensional (2D) method. In this approach, cranial landmarks and their corresponding points on the surface of the skin are marked on an image or screen. The linear distance between two points is then measured using callipers. Later, medical visualisation software has allowed researchers to reconstruct 3D models of heads and skulls from images obtained through MRI, CT, and CBCT [3, 22, 23, 25, 26, 33, 37, 46]. In this three-dimensional (3D) method, digital markers are placed onto the skull and head models at various landmarks. Then, the distance between the two markers is quantified using specialized software, such as Amira, Avizo, OsiriX and 3D Slicer. This approach enables researchers to rotate the 3D skull and head models, examine them from different angles, and individually place the markers on the models [22].

Although various imaging technologies are used in FSTD studies, they differ significantly in terms of image quality and accuracy. Lateral cephalometric radiographs allow for measurements from living individuals in an upright position, reflecting the gravitational effects on facial soft tissue in a realistic manner [22]. However, the measurements can be taken only from the midline landmarks since all structures overlap on a single scan. Besides, subjects are exposed to radiation in this data-obtaining method [6, 38]. In contrast, ultrasound is an efficient, radiation-free imaging modality [6]. It allows measurements to be taken from both midline and bilateral facial points. Stephan et al. [38] described ultrasound as an ideal way for measuring subadults, as it does not expose them to radiation. One significant advantage of ultrasound is that individuals can be scanned in an upright position. However, some researchers have encountered difficulties when placing the ultrasound probe on some cranial points due to the uneven surface of the skull; therefore, the face [47, 48]. Furthermore, the probe compresses the skin, resulting in soft tissue depth distortion. This distortion issue was later addressed by using a stand-off gel pad [22, 38].

MRI and CT are recognised as the most accurate imaging modalities for studying facial soft tissue depth [31, 32]. CT is commonly used for establishing FSTD datasets across various population groups. This method enables scanning living individuals in a non-contact manner. Contrary to radiographs, MRI and CT images consist of a stack of slices, which provide detailed views of structures without overlapping [38]. With the help of 3D visualisation software, it is possible to reconstruct 3D models of the head and skull from CT scans. This allows accurate placement of landmarks on the models, as they can be rotated for better alignment [22]. However, despite these advantages, CT is not considered safe due to radiation exposure. Moreover, soft tissue distortion occurs due to gravity since the individuals are scanned in the supine position [6, 49]. Although there is an upright (or standing) CT system available [50], conventional CT scans remain the standard. MRI is another non-contact and radiation-free imaging modality, but it tends to be more expensive than other options [30, 38]. Besides, the working principle of MRI relies on detecting the behaviour of hydrogen atoms, which are found abundantly in water molecules. Since the skull has a low concentration of hydrogen atoms, MRI fails to generate a precise image of the skull [38, 51]. Furthermore, subjects are scanned in the supine position in MRI as well. Even though there are upright MRI systems available, such as the G-Scan Brio, Stand-Up MRI, and MROpen [52], these relatively new imaging systems are still uncommon. Recently, there has been a rise in FSTD studies utilizing cone-beam computed tomography (CBCT) scans [29, 34, 53]. Research by Vandermeulen et al. [16] indicates that CBCT provides image quality nearly equivalent to that of conventional CT scans. In addition, patients are scanned in the upright position with less amount of radiation exposure. Therefore, the use of this imaging technology could provide significant advantages for the field.

Although there are many comparative studies regarding the inclusion of different imaging modalities in FSTD, there is a lack of research comparing the measurement methods used in FSTD, such as the needle puncture, 2D and 3D. Only a limited number of studies [37, 47, 49] have investigated whether these methods can be used interchangeably and whether the use of different measurement techniques affects facial approximations and recognitions. Therefore, this research aims to explore the potential differences between databases obtained using the 2D and 3D FSTD measurement methods on the same CT images for the first time.

## Materials and methods

The material of this study consisted of the head CT scans of 100 subjects (50 male and 50 female), with an average age of 59 years (ranging between 18 and 99 years). The subjects underwent CT scans for diagnostic purposes at the radiology department of Athens Medical Centre, Athens, Greece. Thus, the subjects of this study were not exposed to radiation specifically for this study. The only exclusion criterion was having any facial condition that may distort facial soft tissue depth.

### CT scan protocol and landmarks

**Fig. 1:**
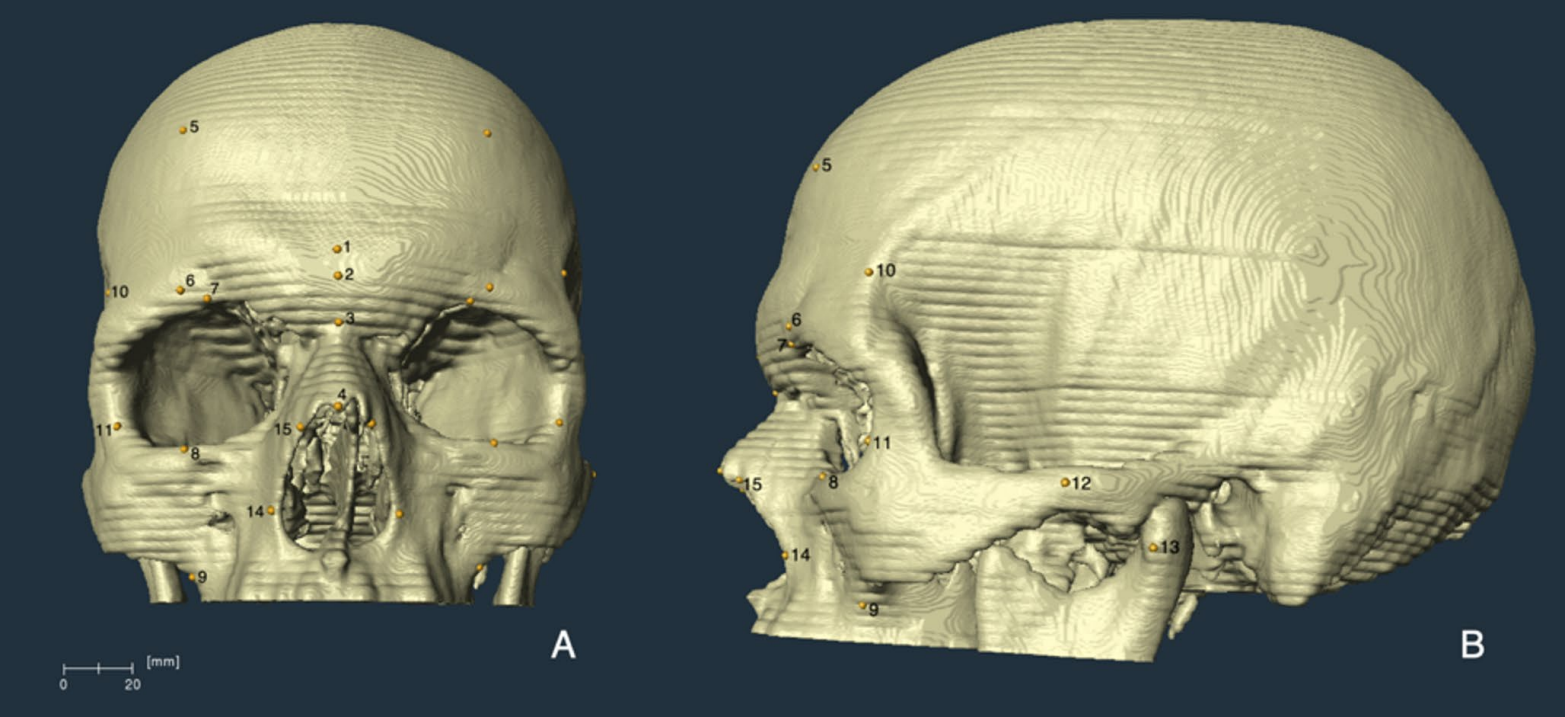
The Frontal (**A**) and lateral (**B**) views of the 3D skull models of an individual, showing the cranial landmarks used for the 3D measurement method. Source: adapted from Coşkun et al. [3]

The CT scans were taken using the Siemens SOMATOM^®^ Sensation 64 (Siemens AG, Forchheim, Germany). The images were recorded following the tube current of 400 mAs, at the tube voltage of 20 kV, with a slice thickness of 2.4 mm and a matrix of 512 × 512 pixels. The subjects were scanned when they were in the supine position. All the images were obtained in the DICOM format (Digital Imaging and Communications in Medicine). A total of 15 (4 mid-sagittal and 11 bilateral) cranial landmarks that can be identified on both 3D skull models and CT slices (Figs. 1, 4 and 5) were selected for this study. Since the radiological examination did not require scanning the whole head, only the upper two-thirds of the skulls were scanned (from the top of the skull to the mastoid processes). Therefore, only those found on the upper portion of the skull were included in this study. The commonly accepted definition of landmarks (Table 1) was obtained from various studies [21–26].

**Table 1:** The definitions of the cranial landmarks that were selected for this study

Landmark	Definition
1. Supraglabella (Sg)	On the frontal bone, 10 mm superior to the glabella
2. Glabella (G)	The most anterior point between the supraorbital ridges
3. Nasion (N)	The midpoint of the suture between the frontal and the two nasal bones
4. Rhinion (Rhi)	The anterior tip of the nasal bone
5. Frontal Eminence (Fe)	Place on the projections at both sides of the forehead
6. Supraorbital (So)	The most anterior point of the supraciliary arch in the axis of the centre of the orbit
7. Supraconchion (Sk)	The most superior point of the orbital rim
8. Orbitale (Or)	The most inferior point of the orbital margin
9. Sub-maxillar Curvature (Smc)	The most supero-medial point on the maxillary inflexion between the zygomaxillare and the ectomolare
10. Frontotemporale (Ft)	The most anterio-medial point of the linea temporalis superior
11. Ectoconchion (Ec)	The most lateral point at the lateral margin of orbit
12. Zygion (Zy)	The most lateral extent of the lateral surface of the zygomatic arch
13. Condylion (Co)	The most lateral point of the glenoid process of the mandible
14. Alare (Al)	The most lateral point on the margin of the anterior nasal aperture
15. Nasomaxillare (Nm)	The most inferior point of the naso-maxillary suture on the nasal aperture

### FSTD measurement methods

**Fig. 2:**
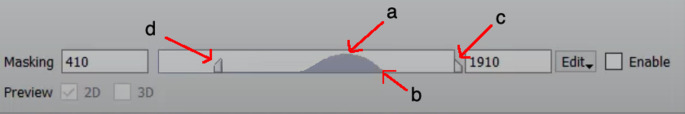
Masking window of the threshold segmentation tool of Amira. The threshold selection of this study was made by dragging the left hand (**d**) to where the histogram mass starts (**b**). The right hand (**c**), on the other hand, was set to the highest possible numerical threshold value

**Fig. 3:**
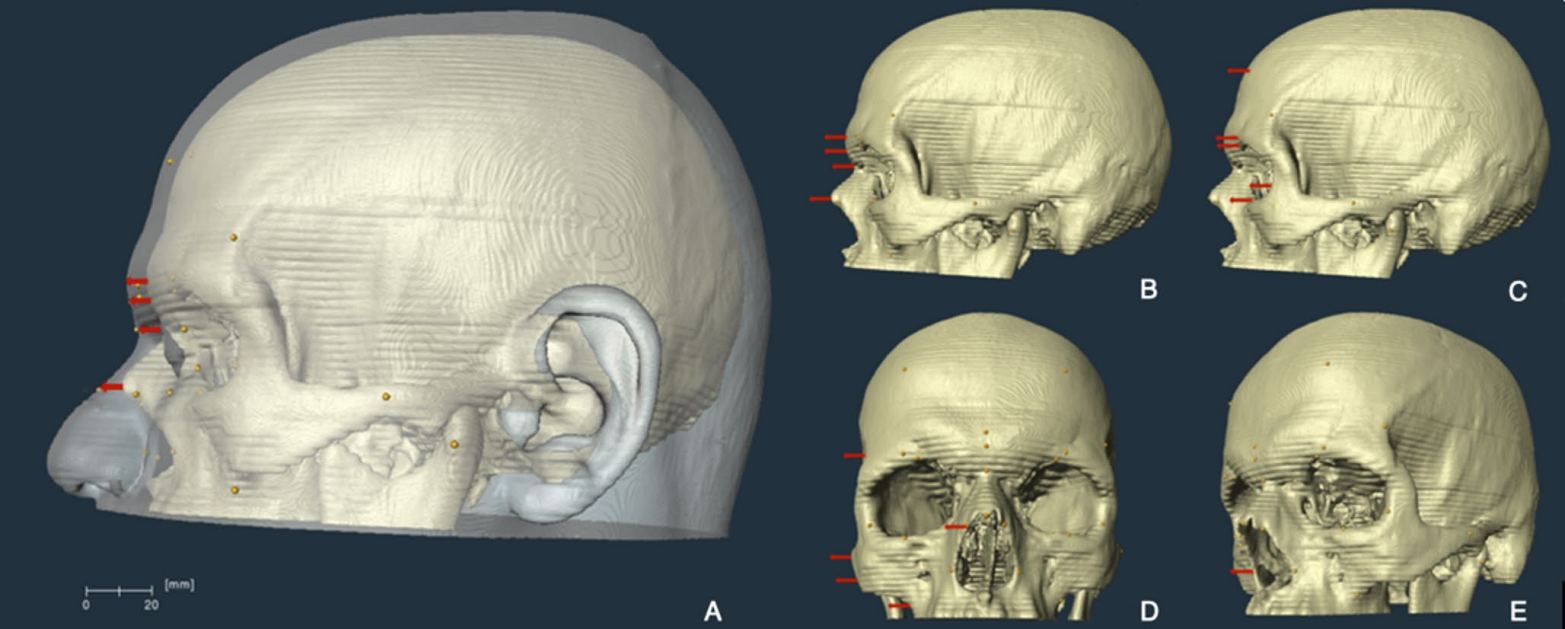
The superimposed 3D skull and head models of an individual (**A**). While the mid-sagittal (**B**) and some of the bilateral (**C**) landmarks were measured when the skull was in the sagittal plane, the rest of the landmarks were measured when the skull was in the coronal (**D**) and/or sagitto-coronal (**E**) planes. Source: adapted from Coşkun et al. [3]

The three-dimensional (3D) measurement method of this study is defined as the quantification of the distance between the cranial and corresponding cephalometric landmarks placed on the superimposed 3D skull and head models (Fig. 3). Before commencing the 3D method, the skulls and heads of the individuals were segmented using the Histogram method [54] in Amira 6.1 (Thermo Fischer Scientific, Cleveland, OH, USA). This method was achieved using the threshold segmentation tool of Amira. Accordingly, the left hand (d) of the masking window was moved to the right until it met the histogram mass (b) (Fig. 2). Afterwards, by visually controlling the image, the left hand was moved gradually and meticulously towards where the histogram made a peak (a) until all pixels of the region of interest (ROI) were selected. The right hand (c), on the other hand, remained as high as possible. The threshold values, also known as CT number or Hounsfield Unit, belonging to the skulls varied between 121 and 146, whereas the threshold values of the head remained between − 703 and − 670.

Before taking measurements, the 3D skull models were positioned in the Frankfurt Horizontal plane (FHP), which implies that a plane passes through both right and left *porions* and both right and left *orbitale* [55]. When the skulls were still in the FHP, the markers were placed on each skull individually by following the descriptions in Table 1. This process was carried out in the sagittal, coronal and sagitto-coronal planes depending on the location of the landmarks on the skull (Fig. 3). Once all the cranial landmarks were placed, the 3D head and skull models were superimposed using the surface generation algorithm of Amira. This algorithm enables the visualisation of surfaces in different styles, such as shaded, outlined, transparent, and points. The head models were created using the transparent surface feature to be able to trace the cranial landmarks with precision (Fig. 3). When the superimposed models were in the sagittal, coronal or sagitto-coronal planes [23], the cephalometric landmarks were then placed on the 3D head models by perpendicularly tracing the cranial landmarks. Subsequently, the linear distance between the cranial and cephalometric landmarks was measured.

**Fig. 4:**
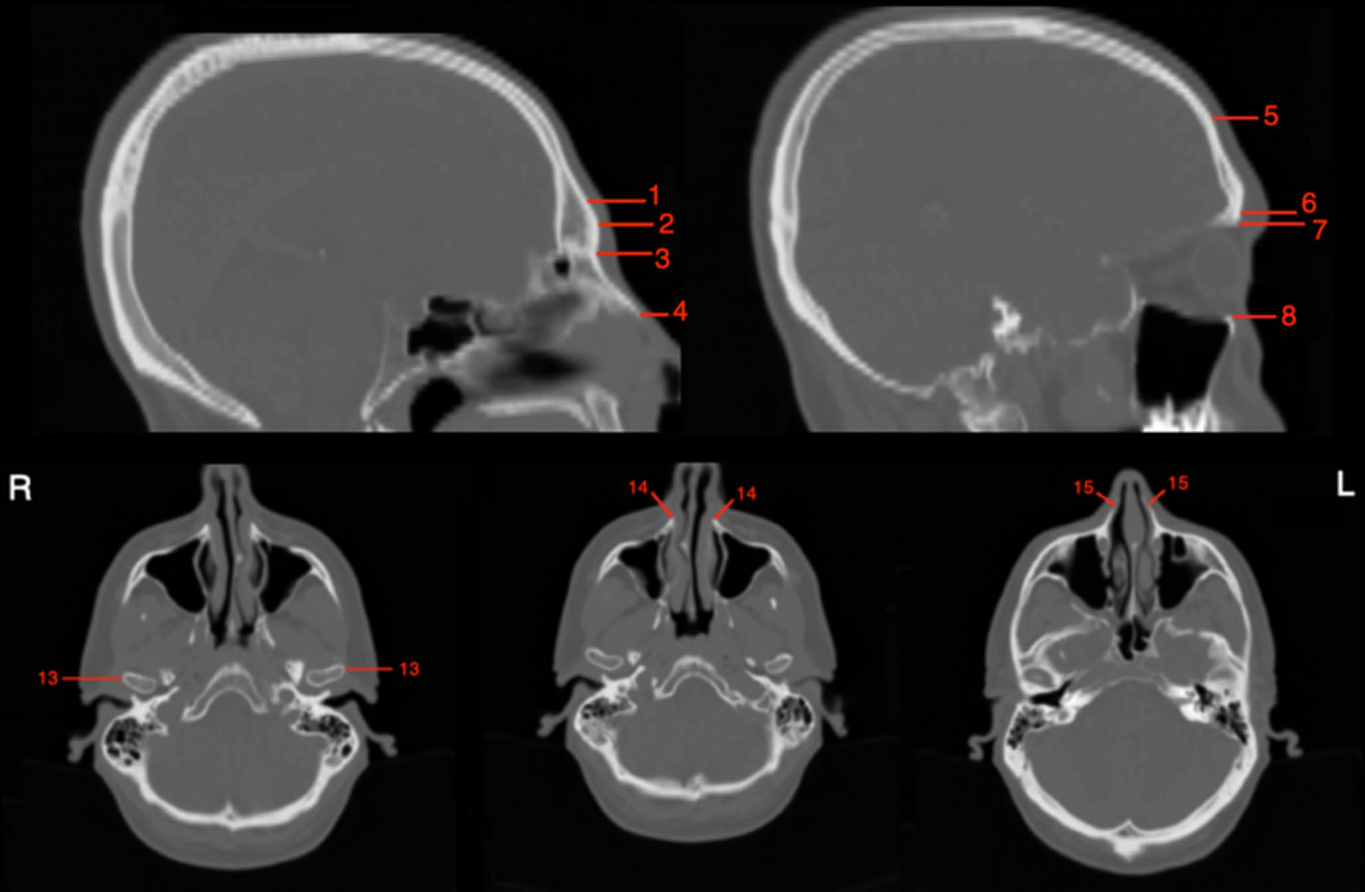
While the sagittal planes of the CT slices (upper row) were used to measure the midline and some of the bilateral cranial landmarks for the 2D measurement method, the transverse planes (lower row) were used to measure the rest of the bilateral cranial landmarks

**Fig. 5:**
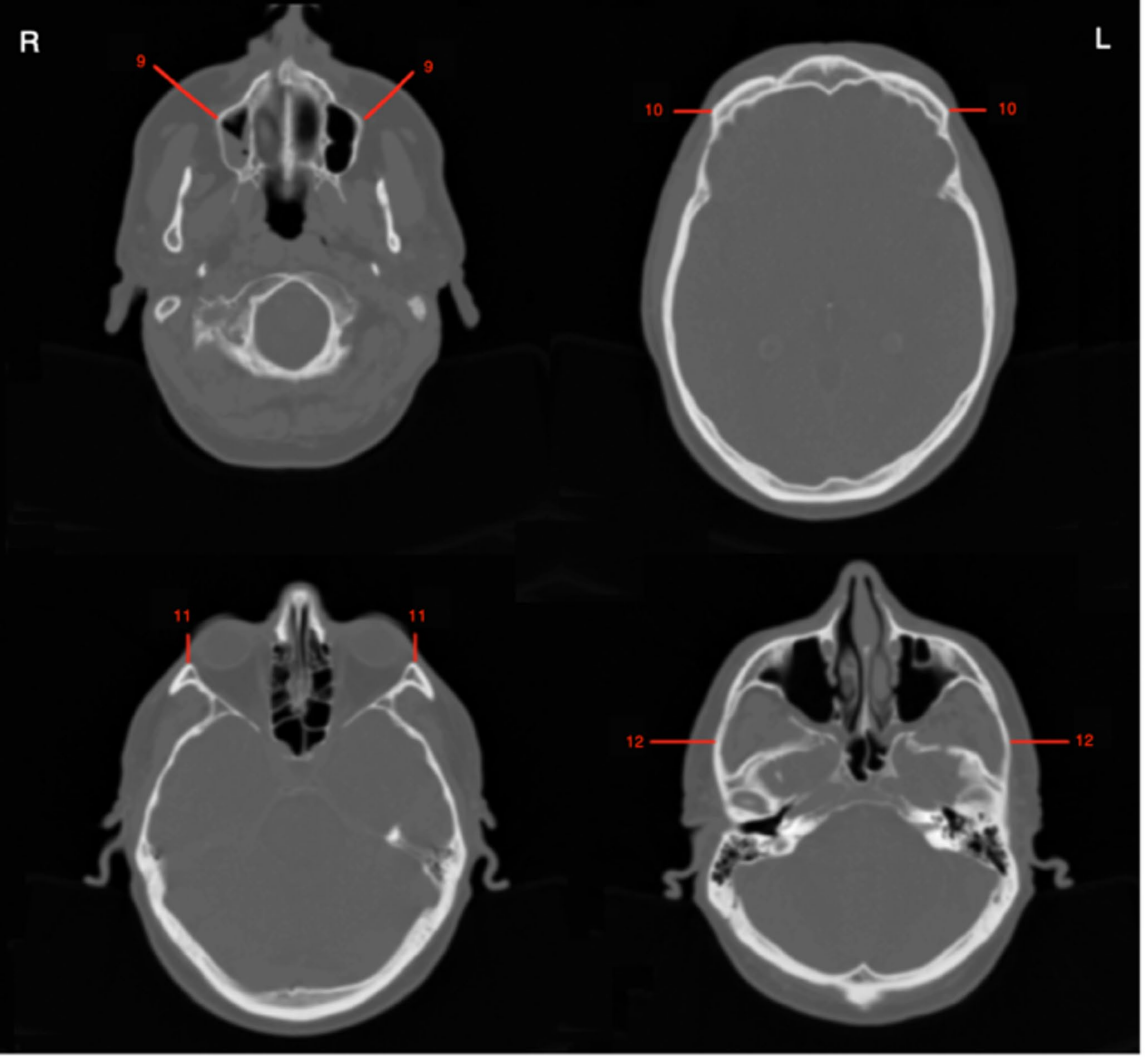
CT slices in the transverse plane, indicating the bilateral cranial landmarks used for the 2D measurement method

The two-dimensional (2D) measurement method of this study is defined as quantifying a linear distance between a cranial and corresponding cephalometric landmark identified on a single CT slice (Figs. 4 and 5). For this method, all CT slices of each individual were examined to identify the location of each landmark. Since Amira does not support placing 3D markers on 2-dimensional CT slices, the 2D measurements were directly obtained from the slices following the same measurement orientation used in the 3D method. In this method, the landmarks were identified and measured only in the sagittal and transverse planes. Positioning the single CT slices in the FH plane was inapplicable. Therefore, the 3D skull models were used for this step as templates. When the 3D models were in the FH plane, they overlapped with the respective CT slice. Then, the skull models were concealed, leaving only the CT slices for the 2D measurement. This process was achieved only for the sagittal plane. The CT slices in the transverse plane could not be positioned in the FH plane due to the technical inadequacy of Amira. All the depth values measured were in millimetres (mm), and they were recorded in an Excel spreadsheet for the statistical evaluations.

### Statistical analysis

Data were analysed using the Statistical Package for Social Sciences (SPSS, version 25.0). The mean facial soft tissue depths were calculated for each sex category. A significance level of < 0.05 was set for the statistical tests used. The distribution of normality was tested using the *Shapiro-Wilk* test. The *repeated measures MANOVA* was employed to evaluate both intra- and inter-observer errors. The comparison between the FSTD datasets of the 2D and 3D measurement methods was made using the *repeated measures MANOVA*. In cases where the data did not follow a normal distribution, a *Friedman* test was applied. Finally, a direct comparison was made between the mean depth values of this study and the reported depth values of the Cretan [56], Czech [33], Slovak [24] and Gujarati [57] populations. The population selection was made among studies that used the same imaging modality and the same measurement methods.

The intra-observer error was assessed by the author through re-measuring the FSTD of 25 randomly selected individuals. The inter-observer error, however, was evaluated by a second observer who had no prior experience with processing CT scans or identifying the cranial landmarks on 3D skull models. Before conducting the measurements, the second observer was given specific instructions. The 3D measurements were taken from models generated by the author. Additionally, these measurements were re-collected from 12 individuals, who were randomly chosen from the original group used to determine the intra-observer error. The measurements were repeated after a four-week interval, following the same protocols for both methods.

## Results

### Accuracy assessment

The results of the intra-observer error assessment showed that all the measurements taken using the 3D method were repeatable and accurate, except for the left *frontotemporale*, *zygion*, *nasomaxillare* and right *alare*. The *repeated measures MANOVA* revealed statistically significant differences in measurements from these four landmarks between the repeated assessments. The results of the intra-observer error calculated for the 2D method were in agreement with those of the 3D method, as most landmarks were re-measured accurately. Only the *nasion* and right *sub-maxillary curvature* exhibited statistically significant differences between the measurements taken (Table 2).

**Table 2:** The results of the *repeated measures MANOVA* conducted for the intra- and inter-observer error assessments of the 3D and 2D measurement methods

	Intra-Observer		Inter-Observer		
Landmarks	Sig. (2D)	Sig. (3D)	Sig. (2D)	Sig. (3D)	
Supraglabella	0.99	0.34	0.59	0.54	
Glabella	0.19	0.21	0.58	0.05	
Nasion	0.01*	0.65	0.21	0.33	
Rhinion	0.17	0.62	0.36	0.07	
Frontal Eminence-L	0.19	0.33	0.24	0.94	
Frontal Eminence-R	0.42	0.32	0.09	0.03*	
Supraorbital-L	0.60	0.96	0.29	0.23	
Supraorbital-R	0.66	0.70	0.41	0.31	
Supraconchion-L	0.94	0.33	0.25	0.13	
Supraconchion-R	0.19	0.08	0.16	0.03*	
Orbitale-L	0.47	0.51	0.05	0.22	
Orbitale-R	0.92	0.35	0.03*	0.29	
Sub-maxillar Curvature-L	0.90	0.56	0.01*	0.40	
Sub-maxillar Curvature-R	*p* < 0.01**	0.57	0.20	0.33	
Frontotemporale-L	0.06	0.03*	0.73	0.03*	
Frontotemporale-R	0.13	0.13	0.55	0.58	
Ectoconchion-L	0.47	0.35	0.77	0.08	
Ectoconchion-R	0.16	0.86	0.29	0.46	
Zygion-L	0.34	0.03*	0.57	0.16	
Zygion-R	0.34	0.91	0.85	0.88	
Condylion-L	0.89	0.22	0.86	0.60	
Condylion-R	0.09	0.80	0.97	0.54	
Alare-L	0.99	0.17	0.62	0.06	
Alare-R	0.71	*p* < 0.01**	0.54	0.98	
Nasomaxillare-L	0.23	0.04*	0.12	0.44	
Nasomaxillare-R	0.31	0.92	0.04*	0.22	

Statistically significant at level * *p*<0.05, ** *p*<0.01

The findings of the inter-observer error were not significantly different from those of the intra-observer error. The results indicated that the measurements of the right *frontal eminence* and *supraconchion*, and left *frontotemporale* were not repeatable for the 3D method. Similar results were observed in the inter-observer error of the 2D method. The measurements taken from the right *orbitale*, *nasomaxillare* and left *sub-maxillar curvature* showed statistically significant differences between the repeated measurements. Therefore, the measurements of these landmarks were considered inaccurate (Table 2).

### Comparison of the FSTD measurement methods

The comparison between the two datasets was made across the four mid-sagittal and 11 bilateral landmarks (Table 3). In males, the mean FSTDs obtained using the 3D measurement method were slightly larger than those of the 2D measurement method in all cases, apart from the left *sub-maxillar curvature*. These depth differences ranged between 0.10 mm and 3.63 mm. The highest differences were noted from the left and right *ectoconchion*, with 3.63 mm and 3.44 mm, respectively. Most of the landmarks showed statistically significant differences in males (Table 3).

**Table 3:** The results of the *repeated measures MANOVA* used for the comparison of the datasets obtained using the 2D and 3D methods

	Male (*n* = 50)						Female (*n* = 50)					
	3D		2D				3D		2D			
Landmarks	*n*	$\:\stackrel{-}{\boldsymbol{\upchi\:}}$	*n*	$\:\stackrel{-}{\boldsymbol{\upchi\:}}$	Diff	*Sig.*	*n*	$\:\stackrel{-}{\boldsymbol{\upchi\:}}$	*n*	$\:\stackrel{-}{\boldsymbol{\upchi\:}}$	Diff	*Sig.*
Supraglabella	50	6.53	45	5.69	0.84	< 0.01**	48	5.93	47	5.10	0.83	< 0.01**
Glabella	49	6.27	50	5.89	0.38	< 0.01**	49	6.03	49	5.44	0.59	< 0.01**
Nasion	50	8.02	50	7.90	0.12	0.06	50	6.76	48	6.42	0.34	< 0.01**
Rhinion ^a^	42	3.30	45	2.84	0.46	< 0.01**	44	2.62	46	1.94	0.68	< 0.01**
Frontal Eminence-L	43	6.34	48	5.00	1.34	< 0.01**	46	5.65	48	4.31	1.34	< 0.01**
Frontal Eminence-R ^a^	45	6.27	48	4.96	1.31	< 0.01**	49	5.55	49	4.23	1.32	< 0.01**
Supraorbital-L	47	9.71	49	8.66	1.05	< 0.01**	48	8.77	49	7.92	0.85	< 0.01**
Supraorbital-R	49	9.75	48	8.92	0.83	< 0.01**	50	8.40	50	7.77	0.63	< 0.01**
Supraconchion-L	50	9.95	50	9.78	0.17	0.34	48	8.94	49	8.67	0.27	< 0.01**
Supraconchion-R ^a^	47	10.03	45	9.90	0.13	1.00	49	8.52	49	8.42	0.10	0.63
Orbitale-L	50	7.18	50	6.32	0.86	< 0.01**	47	6.34	47	5.50	0.84	< 0.01**
Orbitale-R	50	7.40	49	6.60	0.80	< 0.01**	49	6.52	49	5.62	0.90	< 0.01**
Sub-maxillar Curvature-L	40	27.90	38	29.00	−1.1	< 0.01**	36	27.97	35	28.81	−0.84	< 0.01**
Sub-maxillar Curvature-R ^a^	39	29.11	35	29.01	0.10	0.61	36	28.54	35	28.68	−0.14	0.39
Frontotemporale-L	50	6.32	50	5.70	0.62	< 0.01**	48	5.90	48	5.30	0.60	< 0.01**
Frontotemporale-R ^a^	48	6.19	49	5.78	0.41	< 0.01**	50	5.77	49	5.16	0.61	< 0.01**
Ectoconchion-L	49	10.67	50	7.04	3.63	< 0.01**	48	9.66	48	6.35	3.31	< 0.01**
Ectoconchion-R ^a^	45	10.61	49	7.17	3.44	< 0.01**	50	9.56	47	6.36	3.20	< 0.01**
Zygion-L	49	8.71	49	8.36	0.35	0.01*	49	9.79	48	9.32	0.47	< 0.01**
Zygion-R	49	9.10	49	8.76	0.34	0.04*	50	9.89	49	9.33	0.56	< 0.01**
Condylion-L ^a^	50	19.30	50	18.83	0.47	0.57	49	16.51	49	16.55	−0.04	0.66
Condylion-R	50	19.20	49	18.77	0.43	0.02*	49	16.22	50	16.28	−0.06	0.63
Alare-L ^a^	42	9.95	43	8.82	1.13	< 0.01**	43	9.55	43	8.01	1.54	< 0.01**
Alare-R ^a^	39	9.75	43	9.06	0.69	< 0.01**	42	9.23	43	8.11	1.12	< 0.01**
Nasomaxillare-L ^a^	45	5.94	45	4.35	1.59	< 0.01**	34	4.41	48	3.67	0.74	< 0.01**
Nasomaxillare-R	44	6.03	45	4.17	1.86	< 0.01**	33	4.20	47	3.37	0.83	< 0.01**

*n* = number of samples,$\:\stackrel{-}{\boldsymbol{\upchi\:}}$= mean FSTD value

Statistically significant at level *p<0.05, ** *p*<0.01

(^a^) signifies the Friedman test

In females, the mean FSTD values measured using the 3D method were generally larger than those of the 2D method. The mean depth differences between the two methods ranged between 0.10 mm and 3.21 mm. The greatest difference was reported from the left *ectoconchion* with 3.21 mm. In females, most of the landmarks showed statistically significant differences. These results indicated that the FSTDs measured using the two different measurement methods showed statistically significant results.

**Table 4:** The results of the direct comparison conducted between the mean depth values of the present study and those of the Cretan [56], Czech [33], Slovak [24] and Gujarati [57] populations. The FSTD datasets of Cretan and Czech populations were established using the 3D measurement method, whereas the FSTD datasets of Slovak and Gujarati populations were generated using the 2D measurement method

	3D			2D		
Landmarks	Population	$\:\stackrel{-}{\boldsymbol{\upchi\:}}$ (Male)	$\:\stackrel{-}{\boldsymbol{\upchi\:}}$ (Female)	Population	$\:\stackrel{-}{\boldsymbol{\upchi\:}}$ (Male)	$\:\stackrel{-}{\boldsymbol{\upchi\:}}$ (Female)
Supraglabella	Present study	6.53	5.93	Present study	5.69	5.10
	Cretan	5.7	4.8	Slovak	5.1	4.6
	Czech	–	–	Gujarati	4.6	4.3
Glabella	Present study	6.27	6.03	Present study	5.89	5.44
	Cretan	5.9	5.5	Slovak	5.9	5.5
	Czech	6.29	6.01	Gujarati	6.3	5.0
Nasion	Present study	8.02	6.76	Present study	7.90	6.42
	Cretan	7.8	6.7	Slovak	8.0	6.9
	Czech	9.41	8.25	Gujarati	6.4	6.5
Rhinion	Present study	3.30	2.62	Present study	2.84	1.94
	Cretan	3.8	2.6	Slovak	2.5	2.1
	Czech	3.11	2.61	Gujarati	2.6	2.8
Frontal Eminence	Present study	6.34 (L)	5.65 (L)	Present study	5.00 (L)	4.31 (L)
		6.27 (R)	5.55 (R)		4.96 (R)	4.23 (R)
	Cretan	–	–	Slovak	–	–
		6.1 (R)	3.9 (R)		–	–
	Czech	–	–	Gujarati	–	–
		–	–		–	–
Supraorbital	Present study	9.71 (L)	8.77 (L)	Present study	8.66 (L)	7.92 (L)
		9.75 (R)	8.40 (R)		8.92 (R)	7.77 (R)
	Cretan	–	–	Slovak	–	–
		8.6 (R)	7.2 (R)		8.2 (R)	7.2 (R)
	Czech	9.99 (L)	8.91 (L)	Gujarati	7.1 (L)	6.8 (L)
		10.22 (R)	8.93 (R)		7.1 (R)	6.8 (R)
Supraconchion	Present study	9.95 (L)	8.94 (L)	Present study	9.78 (L)	8.67 (L)
		10.03 (R)	8.52 (R)		9.90 (R)	8.42 (R)
	Cretan	–	–	Slovak	–	–
		10.4 (R)	9.3 (R)		–	–
	Czech	–	–	Gujarati	–	–
		–	–		–	–
Orbitale	Present study	7.18 (L)	6.34 (L)	Present study	6.32 (L)	5.50 (L)
		7.40 (R)	6.52 (R)		6.60 (R)	5.62 (R)
	Cretan	–	–	Slovak	–	–
		7.4 (R)	6.2 (R)		7.0 (R)	6.8 (R)
	Czech	8.37 (L)	7.47 (L)	Gujarati	5.4 (L)	5.8 (L)
		8.35 (R)	7.22 (R)		5.1 (R)	6.1 (R)
Sub-maxillar Curvature	Present study	27.90 (L)	27.97 (L)	Present study	29.00 (L)	28.81 (L)
		29.11 (R)	28.54 (R)		29.01 (R)	28.68 (R)
	Cretan	–	–	Slovak	–	–
		29.9 (R)	27.3 (R)		–	–
	Czech	–	–	Gujarati	–	–
		–	–		–	–
Frontotemporale	Present study	6.32 (L)	5.90 (L)	Present study	5.70 (L)	5.30 (L)
		6.19 (R)	5.77 (R)		5.78 (R)	5.16 (R)
	Cretan	–	–	Slovak	–	–
		6.4 (R)	4.6 (R)		–	–
	Czech	–	–	Gujarati	–	–
		–	–		–	–
Ectoconchion	Present study	10.67 (L)	9.66 (L)	Present study	7.04 (L)	6.35 (L)
		10.61 (R)	9.56 (R)		7.17 (R)	6.36 (R)
	Cretan	–	–	Slovak	–	–
		9.4 (R)	7.7 (R)		–	–
	Czech	5.85 (L)	5.85 (L)	Gujarati	6.6 (L)	7.2 (L)
		5.84 (R)	5.87 (R)		6.6 (R)	7.2 (R)
Zygion	Present study	8.71 (L)	9.79 (L)	Present study	8.36 (L)	9.32 (L)
		9.10 (R)	9.89 (R)		8.76 (R)	9.33 (R)
	Cretan	–	–	Slovak	–	–
		9.7 (R)	9 (R)		9.5 (R)	9.1 (R)
	Czech	8.77 (L)	9.19 (L)	Gujarati	–	–
		8.49 (R)	9.17 (R)		–	–
Condylion	Present study	19.30 (L)	16.51 (L)	Present study	18.83 (L)	16.55 (L)
		19.20 (R)	16.22 (R)		18.77 (R)	16.28 (R)
	Cretan	–	–	Slovak	–	–
		–	–		–	–
	Czech	–	–	Gujarati	–	–
		–	–		–	–
Alare	Present study	9.95 (L)	9.55 (L)	Present study	8.82 (L)	8.01 (L)
		9.75 (R)	9.23 (R)		9.06 (R)	8.11 (R)
	Cretan	–	–	Slovak	–	–
		10.9 (R)	10 (R)		–	–
	Czech	12.35 (L)	10.69 (L)	Gujarati	–	–
		12.02 (R)	10.62 (R)		–	–
Nasomaxillare	Present study	5.94 (L)	4.41 (L)	Present study	4.35 (L)	3.67 (L)
		6.03 (R)	4.20 (R)		4.17 (R)	3.37 (R)
	Cretan	–	–	Slovak	–	–
		4 (R)	2.8 (R)		–	–
	Czech	–	–	Gujarati	–	–
		–	–		–	–

The comparison of mean depth values from this study with those from selected studies revealed that the mean depth values for Greek males were slightly larger than those for Cretan males, with differences ranging from 0 mm to 2.03 mm. Notably, both studies recorded the same depth value at the right *orbitale* (Table 4). Similarly, the mean FSTD scores for Cretan females were generally lower than those for Greek females at most landmarks. The largest mean depth difference between the two populations was observed at the *ectoconchion* (1.86 mm), while the smallest difference was found at the *rhinion* (0.02 mm).

When comparing Czech and Greek males, it was noted that the FSTD values for Czech males were higher than those for Greek males at most landmarks, with mean depth differences ranging from 0.02 mm to 4.82 mm. The most significant depth differences were measured at the left and right *ectoconchion*, which were 4.82 mm and 4.77 mm, respectively. In contrast, mean FSTDs for Greek females were larger than those for Czech females, with the largest depth differences recorded at the left *ectoconchion* (3.81 mm) and the right *ectoconchion* (3.69 mm) for females.

The comparison of mean depths scores of this study with those studies that utilized the 2D measurement method revealed similar results to those obtained using the 3D measurement method. The depth values for Slovakian males were slightly higher than those of Greek males, showing variations between 0.01 mm and 0.74 mm. In contrast, the mean depth values for Greek females were generally larger than those for Slovakian females, although the differences did not exceed 1.18 mm. Additionally, the depth values for Greek males were found to be slightly larger than those for Gujarati males, with differences ranging from 0.24 mm to 1.82 mm. On the contrary, the depth values of Gujarati females were higher than those of Greek females, with the highest difference observed at the left *supraorbital*, measuring 1.12 mm.

## Discussions

### Accuracy assessment

Reporting the accuracy and precision of the measurements collected for the anthropometric studies indicates the quality of the study performed [58]. All the measurements taken using the 3D method were subject to skull positioning-associated errors. In Amira, it was impractical to immobilise the 3D models once they were positioned in the FH plane. Each time the skulls were rotated to measure depth, their positions in the FH plane had to be readjusted. This software-related issue might have led to a divergence between the repeated measurements obtained using the 3D method. Additionally, the differences in depth measurements from both methods could be attributed to variations in the identification of cranial landmarks.

The error margin of the measurements obtained using the 2D method was smaller than that of the 3D method. This discrepancy was anticipated due to the use of different measurement methods. Only the sagittal CT slices were positioned in the FHP in the 2D measurement method. Consequently, the measurements collected on the transverse CT slices were free of a potential orientation-related error, unlike in the 3D method. The results indicate that there was low variability in the level of agreement among the observers for both the 3D and 2D measurement methods. Similar findings were observed for both intra-observer and inter-observer reliability.

### Comparison of the FSTD measurement methods

The facial soft tissue depth dataset is a significant component of facial approximation, which provides the metric guide for the creation of faces [59]. Various measurement and data acquisition methods have been used to quantify FSTDs since the first depth measurement study was conducted. However, there is a lack of studies conducted to verify the compatibility of different measurement methods.

The comparison made between the FSTD datasets established using the 2D and 3D measurement methods showed that most of the landmarks indicated a statistically significant difference between the two methods. The 3D method generally measured the facial depths slightly larger than those recorded by the 2D method. However, the absolute depth differences between the two methods were small in most cases, apart from the *ectoconchion*. The depth differences acquired between these two methods might be attributed to several factors, including observer error, inconsistencies in measurement orientation, identification of landmarks, and positioning of the 3D skull models and CT slices in the FHP. Although the same measurement orientations were intended to apply in both methods, the directions might have slightly deviated due to the examiner’s impact and dimensional incompatibilities. Since the 3D skull models made the visual inspection possible, setting the identified measurement directions in the 3D method was more applicable. In contrast, the 2D method was limited in this regard, as each measurement was taken from just one slice (either the transverse or sagittal plane). Moreover, the orientation of the CT slices was slightly oblique. As a result, even if a straight line was aimed between the cranial landmarks and the corresponding cephalometric landmarks, the measurement directions in the 2D method sometimes shifted slightly upward or downward.

Although the measurements were collected by the same examiner, the identification of landmarks varied between different data types (i.e., 3D skull models and CT slices), due to dimensional inconsistencies. The 3D method enabled rotating the skull models and assessing them visually before placing the cranial landmarks. In contrast, identifying the measurement sites in the 2D method was challenging. When the locations of the landmarks were compared by superimposing the skull models with the CT scans, slight variations in the positions of the landmarks were observed. Moreover, the *rhinion* was not measured in four individuals since the tip of the nasal bone could not be reconstructed accurately in the 3D method. However, the *rhinion* of the same four individuals was unknowingly measured in the 2D method. In those cases, the measurements were taken slightly to the right or left of the tip of the nasal bone rather than at the midpoint, as done in the 3D method.

The results of the direct comparison conducted between the 3D dataset of this study and the datasets from the Cretan [56] and Czech [33] population samples were consistent. Small depth variations were calculated. Similarly, the comparisons between the 2D dataset of this study and those of the Slovakian [24] and Gujarati [57] populations showed compatibility. It is noted that studies using the 3D measurement method generally reported slightly higher depth values compared to those utilizing the 2D measurement method. The depth variations observed among these studies can be attributed to several factors, including the impact of different examiners, variations within the populations, the body mass of the subjects, and methodological differences such as variations in measurement directions.

The comparison of datasets in this study showed that the greatest depth variation between the two measurement methods occurred at the left and right *ectoconchion* for both sexes. The direct comparison between the mean depth values of the studies supported this finding. Depth variation of the *ectoconchion* measured within the same measurement method was minimal, with the exception of the Czech population, while the variation measured across different measurement methods was significant. The findings of this study suggested that it is likely to misidentify, therefore misplace, the markers of the *ectoconchion* in the 2D method since the identification of this landmark (Table 1) requires a general evaluation of the skull.

Although the overall FSTD variation across the Greeks and Czechs [33] was slight, the mean depth differences recorded from the *ectoconchion* were considerably high for both sex categories, ranging between 3.69 mm and 4.82 mm. The overall depth variation across the studies can be explained by population variations, different measurement protocols, examiner impacts and most significantly body mass of the subjects measured. The impact of body weight on the FSTD is a well-known fact [22, 60] and a positive correlation between facial depth scores and body mass index was reported by several studies [23, 26, 49]. Additionally, the inspection of the *ectoconchion* identified in both studies revealed that the location of the *ectoconchion* in this study was slightly lower than the *ectoconchion* identified in the Czech study [33]. This finding indicates that a slightly different appreciation of the location of landmarks compromises the comparability between measurements [23, 26].

Kim et al. [37] measured the facial depth of cadavers using the needle puncture and 3D method based on CT scans, using the same measurement sites and directions. This study provides invaluable insight into the influence of measurement direction on the depth scores and the difficulty in identifying the same measurement sites in two different methods. As they concluded, different measurement orientations indeed cause facial soft tissue depth variation. The results indicated that the measurements taken in the 3D method were as accurate as the measurements collected in the needle puncture method. However, they found the needle puncture method less convenient due to the difficulty in identifying cranial landmarks on the skin and the challenges associated with puncturing the skin [37].

Smith and Throckmorton [47] compared the FSTDs obtained using the 2D method on the ultrasound images and lateral radiographs. They reported a small variation in FSTDs between the two datasets, which was explained by difficulties in positioning heads in the same standard position during the collection of ultrasound images and lateral radiographs. This complication was eliminated in this study by using the same CT scans of the same individuals. However, positioning the transverse CT slices in the FHP could not be achieved in the 2D method. This software-related limitation might have resulted in inconsistencies between the measurement orientations of the two methods at the *sub-maxillar curvature*, *frontotemporale*, *ectoconchion*, *zygion*, *condylion*, *alare* and *nasomaxillare*, which were assessed on the transverse slices. The results of the intra- and inter-observer errors (Table 2) support this assumption, as most landmarks that exhibited statistically significant differences in repeated measurements were derived from the transverse plane. Additionally, Smith and Throckmorton [47] explained the depth variation by potential hormonal changes in female subjects, as the ultrasound images and lateral radiographs were collected at different times. In this study, since all subjects were scanned once, this issue was not encountered. However, the hormonal and environmental factors present on the day of scanning, such as air temperature, the phase of the menstrual cycle of the female subjects and the hydration status of individuals, were unknown.

Stephan and Simpson [49] compared the FSTD measurement methods by re-evaluating the reported results of previously published FSTD datasets. They found small depth variations across the studies, which they attributed to differences in the populations studied, various data collection methods (i.e., cadavers, radiographs, ultrasounds, MRIs, and CT scans), and different measurement orientations. In the present study, the potential impact of population differences and different data collection methods was excluded by using the same data for both methods. However, the implication of the same measurement direction was not possible due to dimensional differences between the methods used. Nevertheless, the results of this study aligned with the conclusions drawn by Stephan and Simpson [49], as the mean depth differences observed between the two methods were insignificant.

Although both methods hold their advantages and disadvantages, the 3D method was found to be more effective from the practical point of view. Identifying cranial landmarks and placing them on the skulls was straightforward using the 3D method, as the skull models could be rotated and viewed from any direction. In addition, positioning the skull in the Frankfurt horizontal plane was feasible in the 3D method. Controlling the orientation of measurements was also more convenient in the 3D approach compared to the 2D method. Despite all these advantages, the 3D method was more time-consuming compared to the 2D method since it required segmenting the heads and skulls before taking measurements. Moreover, placing the markers on both sides of the skull and head models, and then collecting measurements between each landmark pair in the 3D method were laborious. Besides, the 3D method requires special software and expertise for segmentation. In contrast, identifying landmarks using the 2D method was more complex. It required experience in examining and identifying the structures on CT scans. In this study, while landmarks were confidently placed on the skull using the 3D method, their positions might have varied slightly in the 2D method. Fewer cranial landmarks can be included in the 2D method compared to the 3D method because some landmarks are identified or placed based on either other landmarks or a general evaluation of the skull. Since markers were not used in the 2D method, measurements could be taken directly, making it more time-efficient. Additionally, the 2D method did not require special software for implementation.

## Conclusions

The availability of the source of data (i.e., cadaver, ultrasound, radiograph, MRI, CT, CBCT), technical equipment for measurements (including needle, computer, software), ethical approval, and the time needed to complete a study are significant factors in determining a measurement method. Hence, the method used may not be purely the choice of practitioners. There are several methods to quantify the FSTD, but only a limited number of comparative studies have been conducted to date. In this sense, this study fills the gap in the field and provides an understanding of the impact of the choice of measurement method. As this study concludes, the 3D and 2D measurement methods using the CT scans provide compatible facial depth results. However, this study found the 3D measurement method more favourable than the 2D method since the 3D method is likely to provide more accurate identification and precise measurements. Despite the advantages of the 3D approach, both measurement methods could be used interchangeably for FSTD studies, taking into account the differences in depth measurements obtained from each method. In a wider context, the results of this comparative assessment could be a reliable reference for FSTD and medical studies.

## Data Availability

The datasets generated during the current study are available from the author on reasonable request.
